# From Lab to Clinic: Artificial Intelligence with Spectroscopic Liquid Biopsies

**DOI:** 10.3390/diagnostics15202589

**Published:** 2025-10-14

**Authors:** Rose G. McHardy, James M. Cameron, David Andrew Eustace, Matthew J. Baker, David S. Palmer

**Affiliations:** 1 Dxcover Ltd., Royal College Building, 204 George Street, Glasgow G1 1RX, UKdavid.eustace@dxcover.com (D.A.E.); matthew.baker@dxcover.com (M.J.B.); 2 Faculty of Clinical and Biomedical Sciences, University of Lancashire, Harrington Building, 135A Adelphi Street, Preston PR1 7BH, UK; 3 Department of Pure and Applied Chemistry, University of Strathclyde, Thomas Graham Building, 295 Cathedral Street, Glasgow G1 1XL, UK

**Keywords:** machine learning, artificial intelligence, vibrational spectroscopy, liquid biopsy, cancer, cancer diagnosis

## Abstract

Over recent years, machine learning and artificial intelligence have become critical components of many cancer detection tests, in particular multi-omic tests such as spectroscopic liquid biopsies. The complexity and multi-variate nature of spectral datasets makes machine learning invaluable in uncovering patterns that enable robust differentiation of cancer signals. However, introducing any AI-enabled medical device into clinical practice is challenging due to the regulatory requirements needed to progress from fundamental research to clinical and patient use. This review explores some of the fundamental concerns in bringing spectroscopic liquid biopsies to the clinic, including the need for explainable artificial intelligence and diverse validation sets. Addressing these issues is essential to accelerate clinical uptake with the ultimate goal of improving patient survival and quality of life.

## 1. Background

Cancer is the second leading cause of death worldwide, with 10 million deaths caused by the disease in 2020 alone [1,2]. One of the primary reasons for the high mortality rates is late-stage diagnosis, which is a common occurrence because symptoms are often absent or non-specific at earlier stages, leading to patients visiting their primary healthcare provider multiple times before receiving a diagnosis. It is known that the cancer stage at diagnosis is highly predictive of cancer mortality, with stage in fact being one of the most important co-factors for the mortality rate within one year of diagnosis in England [3]. This demonstrates the urgent need for early cancer diagnosis in order to improve patient survival.

The current gold-standard for cancer diagnosis is histopathology, in which patient tissue samples are analysed by pathologists to diagnose and stage all cancer types. Despite its wide-spread use, it can be a timely process to return diagnostic results to patients, and histopathology results may vary between experts [4]. The technique also relies on a patient either being referred by their primary care provider, or being admitted to their emergency department, for the tissue sample to be acquired, which can mean early-stage patients are often missed.

Screening tests are one method for catching cancer cases early when present symptoms are not required for further investigation and treatment. Current screening diagnosis routes for patients in at-risk populations include a mammography for breast cancer [5], Pap smear for cervical cancer [6], low-dose Computed Tomography (CT) for lung cancer [7], endoscopic ultrasound for pancreatic cancer [8], and colonoscopy for colorectal cancer [9]. Although many of these methods have been deemed effective, they are often expensive, in some cases invasive, and largely affected by comorbidities [2]. There is therefore an urgent need for a more convenient method for earlier diagnosis for many cancers.

One method that has emerged to overcome these issues for cancer diagnosis are liquid biopsies, a method which describes the collection and testing of bodily fluids distant to the primary tumour site, including blood, urine, cerebrospinal fluid, and saliva. They can identify a wide range of molecular features that can be tumour- or immune-derived that may be indicative of cancer, while being non-invasive and inexpensive when compared to solid biopsies. Although liquid biopsies may miss elements related to the tumour due to the bodily fluids analysed being distant to the tumour site that would be picked up by tissue biopsy, it is often more representative of migratory aspects of the tumour, which can be more aggressive [10].

An additional benefit of liquid biopsies outside of cancer diagnosis is prognosis. As it is a quick and non-invasive method, it increases its repeatability allowing multiple time points to be taken to monitor disease progression, as well as determining if a tumour is resistant to certain therapies or is likely to reoccur [10,11,12].

Although there are commercially available liquid biopsies that specifically look into single biomarkers, there are fundamental limitations with these tests. As an example, many liquid biopsies aim to measure the levels of Circulating Tumour DNA (ctDNA) and Cell Free DNA (cfDNA) within the blood stream. cfDNA are degraded DNA fragments released by cells into bodily fluids, while ctDNA are the portions of this cfDNA that are specifically released by tumour cells [13]. While ctDNA can provide information directly from the tumour, it is often found in very low amounts, particularly in early-stage tumours. To put into context, ctDNA makes up only 5–10% of the total cfDNA in early-stage disease. This illustrates why the measurement of ctDNA cannot be relied upon for early-stage disease, which is where liquid biopsies are required the most [12].

There are also a number of factors that can cause significant variations in cfDNA levels, and thereby ctDNA levels. Aging is one of the largest confounding factors in which cfDNA levels can increase, due to the increase in somatic mutations in non-cancerous lesions [11]. The level of cfDNA in the bloodstream can be highly variable; day-to-day variation in cfDNA levels have been estimated to be almost 25%. This means it can be difficult to determine the significance of the changes in cfDNA levels [14].

There are many tests available that look into the analysis of a single biomarker, for example CA 19-9 for the diagnosis of pancreatic cancer [15]. However, even though this is a widely utilised test, CA 19-9 has a particularly poor sensitivity in symptomatic patients, as well as not being a universally applicable biomarker since it is related to the Lewis blood group antigens, which not all patients express [16].

The analysis of a single biomarker has proven not to be as successful as the analysis of multiple biomarkers, which has been demonstrated widely across the literature [17,18,19,20,21,22,23,24,25,26,27,28,29,30,31,32,33,34,35,36,37,38,39,40,41,42,43,44]. Biomarker levels can vary across patients depending on an array of confounding factors, such as genetics and diet. Therefore, analysing multiple markers within a combined biological signature may enable more robust analysis [11,45]. This is where spectroscopic liquid biopsies that utilise techniques such as Attenuated Total Reflectance (ATR)-Fourier Transform Infrared (FTIR) spectroscopy have addressed some of the intrinsic limitations of current liquid biopsies.

While genetic-based tests focus on information produced by the tumour itself, FTIR spectroscopy allows a global biological signature to be attained, collecting information from the tumour itself as well as from the body’s response to the tumour [46]. It is also a non-invasive and non-destructive technique that is highly sensitive to molecular changes [47].

The use of FTIR spectroscopy for liquid biopsies has been widely reported in the fields of bladder cancer [48], brain cancer [49], ovarian cancer [50], colorectal cancer [51], and lung cancer [52], as well as other cancers and diseases. Although the background of FTIR spectroscopy applied to biological applications is beyond the scope of this review, if interested, readers can find these details in Baker et al. [53].

FTIR spectroscopy is able to capture multiple tumor and non-tumor derived biomarkers within one measurement of the blood [46,54,55]. As the blood comes into contact with every organ of the body, it is able to provide information on a range of biomarkers. Although tumor derived markers, such as ctDNA, provide information related directly to the tumor, they are often found in low, almost undetectable amounts during the early stages of tumorigenesis. However, non-tumor derived makers, originating from the immune response to the tumor, are often easier to detect than tumor-derived markers during the early cancer stages [56]. FTIR spectroscopy is able to detect several of these markers in the blood, from proteins structures such as amino acids and nucleotides, to carbohydrates structures, such as glucose. Nucleotide and DNA changes can also be picked up from the analysis of phosphate and lipidic groups, which are key in detecting tumor-related changes.

In the past 30 years, Machine Learning (ML) and Artificial Intelligence (AI) has been utilised for multiple diagnostic tests, particularly within cancer diagnostics [57]. Cancer produces complex signals which can be detected using multiple analytical methods that produce data in different modalities. ML analysis is therefore vital to find patterns in complex data, even those from differing modalities [12].

ML has demonstrated successful use within cancer diagnostics, including the ML analysis of Magnetic Resonance Imaging (MRI) images [58,59,60], CT images [61,62,63], and mammography images [64,65]. These applications of ML in imaging diagnostics spurred research towards the direction of combining ML with the much cheaper method of spectroscopic liquid biopsies.

**Figure 1:**
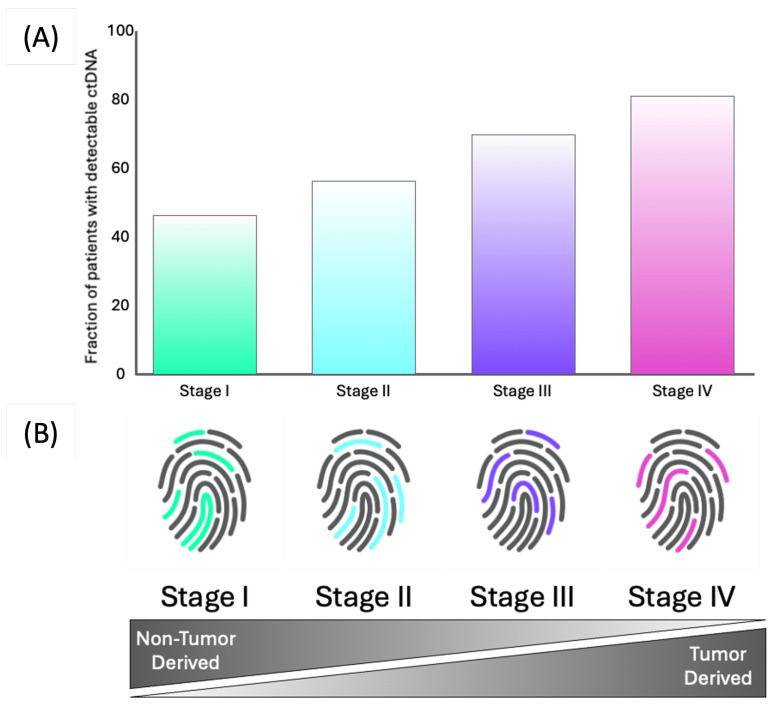
The difference in model sensitivity when using only tumor-derived information, compared with using the IR spectral signature as ML features. (**A**) The fraction of detectable ctDNA in patients. Adapted from [67]. (**B**) Different contributions of tumor and non-tumor-derived information across the stages gives each stage a different spectroscopic fingerprint.

**Figure 2:**
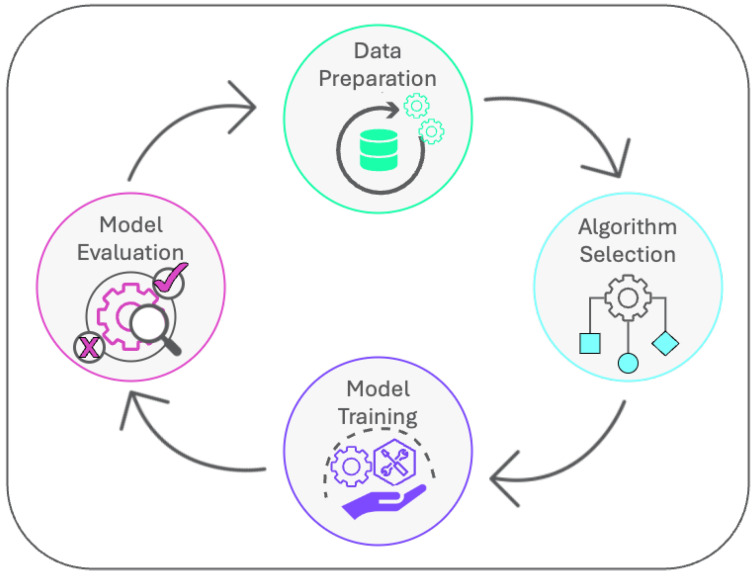
Machine Learning life cycle.

Combining ML with spectroscopy is particularly useful due to the complexity and multivariate nature of Infrared (IR) spectra. In order to relate this data to a cancer diagnosis, multivariate analysis is needed to uncover these complex data patterns [4]. Just one example of this is from Sala et al., who were able to utilise machine learning and FTIR spectroscopy in a proof-of-concept study to diagnose pancreatic cancer from healthy controls with a sensitivity and specificity of 92% and 88%, respectively [55]. Noticeably, the use of the spectroscopic signature as features for an ML model has an impact on creating an equal sensitivity across the cancer stages, compared to using only tumor derived information, where the sensitivity decreases as the cancer stage decreases. This was demonstrated in a multiple cancer test by Cameron et al. and is further illustrated in Figure 1 [66]. Figure 2 demonstrates how the level of detectable ctDNA is much lower in early stage cancer cases, which emphasises the need to measure non-tumor derived information. The different ratios of tumor and non-tumor derived information across the cancer stages creates a unique spectroscopic fingerprint that allows the equal prediction performance shown in the work by Cameron et al. [66].

Although this demonstrates just one example of the promising results that can be obtained from combining ML with spectroscopic liquid biopsies, this technology is still in development and is not yet in routine clinical use. There are many barriers that have slowed the translation of these tests into clinical practice, including data availability, as well as lack of guidelines and regulations provided for the development of AI diagnostic tests.

There are reviews within the literature that discuss ML use within cancer diagnostics [10,11,12,14,45,68]. as well as reviews that look into the use of ML with IR spectroscopy [47,69,70]. However, to the best of our knowledge, there is only one review article by Sattlecker et al. in 2014 that looks specifically into the use of ML for IR spectroscopic liquid biopsies [4]. Although the article by Sattlecker et al. goes into detail surrounding the use of ML alongside IR spectroscopy within cancer diagnostics, it primarily looks into tissue biopsies, and it provides little detail about the regulatory standards these tests should meet. Moreover, it is more than a decade old, and therefore misses recent developments in ML and spectroscopy. The hurdles that these tests need to overcome to progress from academic research to clinical use has also not yet been covered. This review aims to fill these knowledge gaps to illustrate which standards are currently in place for ML within spectroscopic liquid biopsies, as well as what is required in the future for the field to advance.

## 2. Model Development

Once a diagnostic problem has been identified, and suitable clinical and analytical data have been acquired, the development of a diagnostic model involves four main steps: data preparation, algorithm selection (including hypothesis establishment), model training, and model evaluation, as shown in Figure 2. Depending on the project in question, model deployment can also be the last step in this cycle. The details involved in each of these steps will be discussed in the following sections.

### 2.1. Data Preparation

Before any ML models can be trained, data, especially spectral data, must be preprocessed. Preprocessing is required to improve the signal-to-noise ratio by eliminating unwanted signals caused by the instrument or the environment that therefore can be removed as they have no relation to the analyte of interest. These unwanted signals can relate to fluorescence, calibration errors, stray reflections or IR light, and laser power variation [47,71]. As ML models cannot differentiate patterns caused by instrument differences from a biologically relevant signal, preprocessing is a fundamental step before model training and has been shown to significantly improve model performance [68]. It is important to remove these unwanted signals to ensure the model will make diagnostic predictions on future samples, rather than classifying samples based on instrumental or environmental factors. The in-depth theory of the various preprocessing methods, such as normalisation and ExtendedMultiplicative Signal Correction (EMSC), will not be discussed, but the overarching themes with relevant references where more information can be found are in Table 1.

A relatively new method of preprocessing spectra is to use Deep Learning (DL). DL has the ability to automatically extract features of the spectra without explicit human intervention. This allows DL models to be less reliant on prior expertise about spectral noise as well as being able to eliminate these distortions simultaneously [47].

There are many examples of the use of DL in the literature for spectral preprocessing. Raulf et al. were able to utilise a neural network to correct Mie scattering within IR spectra. This neural network was used as a regression model with a raw FTIR spectrum as input, and then used to approximate the Mie scattering and hence correct this scattering to produce a Mie scattering corrected spectrum. Not only were they able to replicate the corrections that would be made by EMSC, they were able to make the corrections using the neural network at a fraction of the compute time. However, the study was able to identify its own limitations in that the model could only correct spectra that are similar to the training spectra, making it non-generalisable [78].

Guo et al. expanded this by using a U-shaped convolutional neural network to preprocess spectra by taking a spectrum as input and transforming these to the corrected absorbances. By simulating their training data, they were able to introduce multiple spectral distortions to improve the quality of model training. When tested on an external test set that was not augmented, they were able to successfully preprocess the spectra to the same ability as Raulf et al., yet simultaneously demonstrating the transferability of the model [79].

As there are many preprocessing techniques available, as well as many combinations of the order of the preprocessing steps, DL can offer an alternative to a trial-and-error approach to spectral preprocessing seen previously within research [72].

### 2.2. Algorithm Selection

There are four main categories of machine learning algorithms, of which the first two are currently most relevant for spectroscopic liquid biopsies: supervised and unsupervised learning, semi-supervised learning, and reinforcement learning. Unsupervised learning involves the input of data into a model that have no output labels associated with them. The aim of this type of machine learning is to find structures and relationships with the data. A common form of unsupervised learning is known as clustering. This involves separating the data into clusters, which, in theory, share some kind of properties or characteristics. Unsupervised methods have the benefit that they can discover hidden patterns and groupings within unlabelled data without any human intervention [80].

On the other hand, supervised learning involves training a machine learning model with data that has already been labelled. During training the model learns relationships between the descriptors (features) and the correct output labels. The trained model may then be used to predict labels from the descriptors for new data.

For supervised learning, there are two different tasks that can be carried out. If the output values for the data are continuous, then this is a regression task. However, if the output values are discrete, then this is a classification task [81]. The selection of the machine learning algorithm is therefore dependent upon the problem output labels. The theory of the various supervised learning techniques (such as Random Forest (RF) and Partial Least Squares (PLS)), as well as unsupervised techniques, have been covered previously and are outside the scope of this review [82].

Recently, deep learning DL—a sub-field of machine learning—has exploded driven by advances in computer hardware and the arrival of big data [83]. DL was developed to solve the problems that standard machine learning models have when trying to recognise speech and objects, by training models on high-level abstractions of data [84].

DL primarily refers to the Neural Network (NN) architecture. Deep NNs can be considered as NNs with many hidden layers [85]. Before rceent developments in computational software and hardware, including the use of Graphical Processing Units (GPU)s, standard NNs could only afford to have one or two hidden layers. DL now allows multiple hidden layers in order to cope with large volumes of data [86]. Again, the theory of these neural networks have been covered elsewhere and will not be discussed in detail within this review [84].

When choosing the optimum algorithm, there is a balance between ML performance and interpretability. Although the more complex DL algorithms can produce better predictions, they also produce a black-box effect in which the underlying patterns within the data are unknown. This leads to a lack of trust in algorithms, whereas the more traditional ML methods can be easier to interpret [87]. The methods to evaluate a model’s performance will be discussed further.

### 2.3. Model Training

When validating the performance of the machine learning model, the ability of the model to fit perfectly to the training data is not enough for it to be applicable for real-world use. The model has to be tested on a set of data that the model has not seen before. The simplest way to do this is to split the dataset into three groups: the training, validation, and test set. The training set has its outputs known to the model and is used to help the model learn patterns within the dataset. The validation set is used to determine the prediction error of the model and to tune the appropriate parameters. Finally, the test set is used to determine the overall performance of the model as a set of samples that have not been seen before by the model [82].

If the model fits very well to the training data, but fails to accurately predict unseen testing data, this is often an indication of over-fitting. Under-fitting is the opposite issue where the model does not fit to the training data very well and also performs poorly on the test set [88].

For small datasets in particular, using a single test set can result in sampling bias, whereby the performance depends on the choice of data points. It is good practice to repeat model training multiple times using different splits of the data into training, validation, and testing sets, to enable the mean and standard deviation of the performance metrics to be reported.

Cross-Validation (CV) is one method of repeating model training to reduce sampling bias. It can be known as *k*-fold CV where *k* is the number of folds involved. For example, in a 5-fold CV, the dataset is split into five separate equally sized folds. For each CV, one of these folds is used to validate the model, while the model is built on k − 1 folds combined. This is repeated for all folds and the average performance across all folds is used as the overall performance of the model.

Another method to reduce sampling bias is resampling (or Monte Carlo CV). Resampling allows an initial shuffle of the training and test sets, and then for each consecutive resample, a new train and test set is created. This mixing of samples for each resample allows the reduction in sample bias by changing the samples the model is trained and tested on each time. Model performance metrics can then be averaged across all resamples to give an overall picture of the model. Another commonly used variation of this idea is bootstrap averaging (“bagging”) [89].

If a single training and validation set split is used to tune the hyperparameters in the ML model (e.g., the number of components in a PLS model), the optimisation may also be susceptible to sampling bias, with the result that the model trained with the selected parameters does not generalize to other datasets. This can be addressed by tuning the hyperparameters to optimise a cross-validated metric.

**Figure 3:**
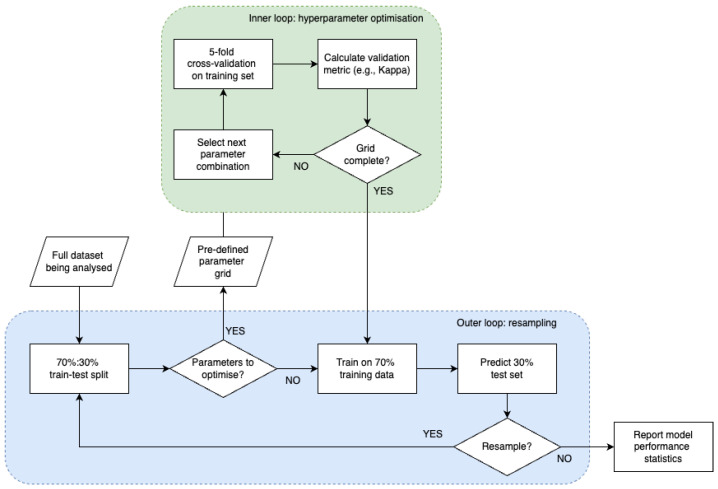
Example of nested CV process.

Given the need to repeat both model training and model testing to avoid sampling bias, an efficient experimental design is nested cross-validation, which also prevents data leakage by ensuring that the test set is not used in model training or optimisation. Figure 3 demonstrates an example workflow of nested CV.

### 2.4. Model Evaluation

#### 2.4.1. Performance Metrics

When determining the model performance, many different parameters need to be considered. As this review is focused on the classification of patient samples for diagnostic purposes, only classification metrics will be covered. Most classification algorithms can output predictions as both class labels (e.g., cancer or non-cancer) and class scores (which equate to a probability, of e.g., cancer, when correctly calibrated). The class labels are obtained by applying a threshold to the class scores. For example, if the threshold is 0.5, predicted scores of 0.4 and 0.6 would be assigned negative and positive class labels, respectively. By default most algorithms use a threshold of 0.5, but this can be tuned to modify the ratio of predictions of each class to optimise the algorithm for use in different healthcare settings. Different metrics are used to quantify the accuracy of the predicted labels and scores. The metrics in the former group can be expressed as either fractions or percentages. In terms of binary classification, the simplest metric is the fraction of predictions that are correct. This is referred to as “accuracy”, and can range from 0 to 1, with 1 representing perfect classification.

However, accuracy can be misleading for imbalanced datasets. For example, in a dataset comprising 100 samples of which 90 are in the positive class, the accuracy of a model that only produced positive predictions would be 90%, which is not a true representation of the performance of the model. For this reason, other performance parameters need to be considered, which are often derived from a confusion matrix consisting of true positives (True Positive (TP)s) and true negatives (True Negative (TN)s), which are the number of correct positive and negative predictions, respectively, and false positives (False Positive (FP)s) and false negatives (False Negative (FN)s), which are the number of incorrect positive and negative predictions, respectively. By convention the disease state (e.g., cancer) is normally taken as the positive class. Using this confusion matrix, performance parameters such as sensitivity, specificity, Positive Predictive Value (PPV), and Negative Predictive Value (NPV) can be determined at certain thresholds to give the overall performance of the model.

Sensitivity, sometimes known as recall, is the proportion of actual positive cases that are correctly classified by the model. This again ranges from 0 to 1, with 1 representing perfect classification. Specificity is the proportion of actual negative cases that are correctly classified by the model, again ranging from 0 to 1, with 1 representing perfect classification. PPV, also known as precision, is the fraction of cases that were predicted as positive that are actually positive, ranging from 0 to 1, with 1 representing perfect classification among positive samples. NPV is the opposite in that it represents the fraction of the cases that were predicted as negative that are actually negative, ranging from 0 to 1, with 1 representing perfect classification among negative samples.

In particular for medical diagnostics, the pre- and post-test probability are important for determining the usefulness of a liquid biopsy test. The pre-test probability is the likelihood that the patient has the disease before the test result is known. It is often taken to be the disease prevalence in the target population. The post-test probability is the likelihood that the patient has the disease after the diagnostic test result is known. It takes into account both the pre-test probability and the accuracy of the test [90].

The pre- and post-test probability metrics can give a clear insight into how a test would perform in a clinical setting. For example, although a study may perform well with a 50% disease prevalence in a case control study, the post-test probability would be expected to decrease significantly in a real-world population where the disease prevalence is much lower.

The majority of ML models output scores that when calibrated can be considered to be probabilities. These scores can be calibrated using Platt scaling, which fits a logistic regression model to the model scores to produce a probability distribution [91]. Based on either the scores or the probabilities, the user picks a threshold to define positive and negative. Most liquid biopsies provide a positive or negative classification rather than a disease probability; the former is often preferred in practice to avoid subjective clinical decisions.

The performance metrics discussed thus far are all known as threshold-dependent metrics, meaning their value depends upon the user choosing a probability threshold in which to make predictions. Therefore, these metrics can change dependent on the application of the model.

Threshold-independent performance metrics can be used to compare multiple models. One way to do this is to use a Receiver Operating Characteristic (ROC) curve to visualise the performance of the model by plotting the sensitivity versus 1—specificity at a range of probability thresholds. This ROC curve aids in the tuning of the probability threshold to fit the application of the model [92].

**Figure 4:**
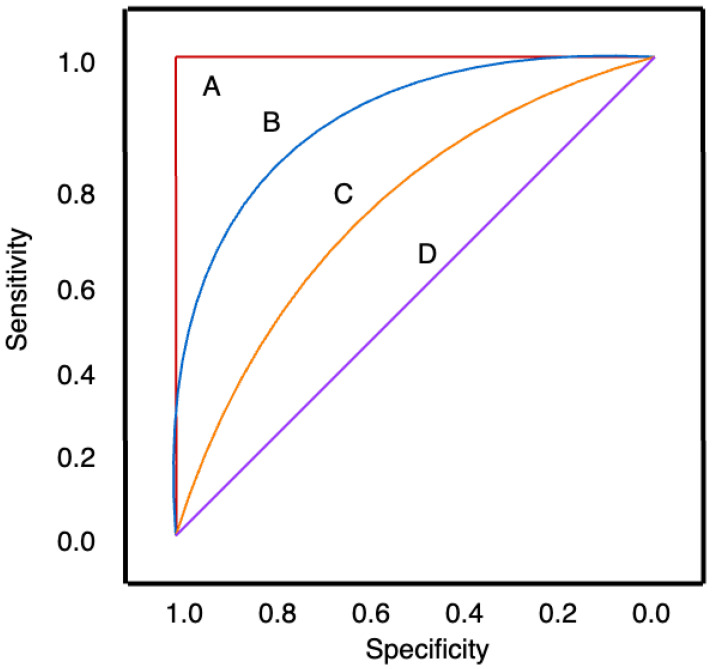
Example ROC curve. (A) A perfect classifier with an AUC of 1. (D) Has an AUC of 0.5, representing a random classifier. (B, C) have AUCs that lie between these extremes, with (B) having a higher AUC than (C) and therefore a better overall model performance. Adapted from [93].

From this ROC curve, a singular metric can be acquired, known as the Area Under the Curve (AUC), which measures the area under the ROC curve. AUC ranges from 0 to 1; a model with 100% incorrect predictions would result in an AUC of 0, while a model with 100% correct predictions would result in an AUC of 1. An AUC of 0.5 represents a model that has a predictive ability no better than random guessing. This can be seen in Figure 4 [92].

Other threshold-independent metrics include the area under the precision–recall curve, which plots precision versus recall for different thresholds. However, as it uses precision and recall, it does not make use of the true negatives. It, again, would have an area under the precision recall curve of 1 for a perfect classifier, and an area of 0.5 to represent a random guessing classifier. Another metric is log-loss, which looks at how close the prediction probability is to the actual value. Log-loss can range from 0 to infinity. If the log-loss is 0, then this signifies that there is no divergence between the predicted probability and the actual value. The higher the log-loss, the more divergence there is.

#### 2.4.2. Challenges in Model Performance Evaluation

Metrics that are threshold-independent can often lead to incorrect clinical conclusions of the model. As an example, although AUC provides an overall metric to describe the model, it does not provide any information on how the model will perform at a chosen clinical threshold, i.e., whether it will perform at different disease prevalences [12].

Most ML metrics reported in the literature do not account for disease prevalence and therefore cannot represent the probability a patient who is diagnosed as cancer actually has cancer. PPV and NPV are important metrics to report in this regard. Even though a model’s sensitivity and specificity may seem good, when used as a screening test on a general population where the prevalence will be extremely low, the post-test probability will also be low. The post-test probability will therefore be impacted by the target population of the ML diagnostic test. However, care must be taken when interpreting PPV and NPV values in the literature, especially when they are reported for datasets with higher prevalences than those that would be observed in clinical use [94].

As discussed previously, threshold-dependent metrics can also be impacted by class imbalance in the dataset. For example, if the cancer prevalence in a dataset was only 5% and the ML model classified all samples as non-cancer, then the model would report an accuracy of 95%, yet the PPV would be zero [95].

Thought should always be given to the consequences of tuning the probability threshold. Even if a threshold is chosen to minimise the number of false negatives, considerations should be given to the subsequent increase in false positives, which can lead to patients undergoing unnecessary invasive testing and causing undue stress [12]. The threshold would be different, for example, for patients that require possible referral to further non-invasive tests or those that require possible referral to more invasive studies, such as tissue biopsies. Overall, in a triage or symptomatic population, a lower threshold is often chosen to tune for high sensitivity. Whereas in a screening or asymptomatic population, a higher threshold is normally chosen to tune for a higher specificity [96].

Overall, various metrics should be calculated when attempting to evaluate ML models, both threshold-dependent and threshold-independent. A single metric is not enough to encompass the entire performance of the model, and the clinical task and target population should be known beforehand to determine if the classifier is successful [94].

ML performance often differs between proof-of-concept and prospective studies. Proof-of-concept, or retrospective studies, are key for ML development to determine whether the model has potential clinical use. However, they often produce elevated performance metrics in comparison to prospective studies in which the model may encounter samples outside its applicability domain [97].

Even though overall model performance metrics provide a useful picture of the diagnostic test, they allow no insight into the generalisability of the model. If the model performs well during proof-of-concept studies, there is no guarantee that the model will be able to predict samples outwith the data it was trained on. This can also be known as out-of-distribution uncertainty and is caused by population differences between datasets. It is often something that is overlooked when ML is applied to healthcare, with McDermott et al. reporting that only 23% of papers have datasets collected from multiple institutions [98]. It can be reduced by introducing more data during model training and preprocessing data to amplify consistent signals and reduce unwanted noise.

## 3. Challenges and Opportunities of Applying ML and AI to Spectroscopic Liquid Biopsies

### 3.1. Challenges

Despite the many opportunities associated with ML for spectroscopic liquid biopsies, there are inherent challenges that have made it difficult to fully translate these techniques into clinical practice.

As described previously, although DL methods are growing in popularity, their complex structures require large training sets in order to avoid overfitting, causing them to be unsuitable for most liquid biopsies [45]. Clinical data can be expensive and laborious to acquire, so it can seem that simpler models are outperforming DL models even though dataset size is the limiting factor [4]. Even when large volumes of data are available, the data must be of high quality, as even advanced DL algorithms cannot establish generalisable relationships in excessively noisy data [45].

Despite the increased use of ML within liquid biopsies, the advancement of these techniques has plateaued and failed to keep up with the latest AI technologies. This is thought to be because of ML expertise within the liquid biopsy field not matching that of other industries [10].

One of the main reasons for absence of clinical uptake of liquid biopsies that utilise ML is the lack of interpretability. Even when the performance of the model is shown to be highly effective, it is often difficult to understand the underlying relationships that the model is using to make the predictions [45].

Unlike traditional statistical methods, which are much easier to understand as they rely on prior knowledge and few clinically important features, ML relies on complex correlations between large numbers of features, which results in a black-box effect of being unable to explain how a prediction has been made [99]. This makes the implementation of ML liquid biopsies difficult due to the lack of trust from clinicians towards the test [12]. When decisions made based upon liquid biopsies can impact patient outcome and care, it is important that the medical professional understands the prediction, so that this can also be explained to the patient [100].

Another limitation of ML within liquid biopsies is the effect of analytical cofounders. Instrumentation, batch effects, and between-site variability can cause unwanted differences between samples. If the relationship between ML features and diagnostic outcomes is not well-understood, there is potential that successful predictions could be made based upon confounding factors unrelated to the cancer biology [14]. ML methods can also unintentionally learn correlations between patient demographics, causing bias in predictions towards certain populations [12]. This algorithmic bias needs to be identified to understand the limitations of a liquid biopsy test, as well as ensuring models are trained on diverse patient datasets [100].

Technologies using ML are also limited to the benchmark tests they are based upon. Supervised ML requires labelled samples and in the case of biopsy samples, most samples are labelled based on histopathology diagnosis. This results in a ceiling in ML performance being as good as histopathology, which can vary due to differences in expert opinion. To resolve this, patient follow-up should be required to ensure correct diagnosis of samples and to therefore improve overall model performance [4].

For models to be adapted successfully into the clinic, it is important for the model to be generalisable to a diverse patient population. It is essential that they are trained and validated on clinically relevant data, as the use of non-clinically relevant datasets can lead to overestimation of the model’s performance. For example, this can occur when the training and validation sets are not representative of the range of pathologies encountered in clinical usage. Despite validation strategies, such as k-fold cross validation, providing more robust methods to determine the performance of the model, they do not factor in different patient populations and clinical settings. In particular, the complexity of spectral data can cause further overfitting to specific datasets [101].

### 3.2. Opportunities

There are many benefits associated with the coupling of ML with spectroscopic liquid biopsies. The primary benefit is ML’s ability to learn from highly complex data often produced by spectroscopic analysis.

Cancer sample data can be high-dimensional and non-linear. For liquid biopsies in general, the complexity of datasets mean that traditional statistical methods struggle to identify patterns within the heterogenous data [10]. ML benefits primarily from being able to analyse this type of data, which explains why its use within cancer diagnostics has grown exponentially [11,102]. Traditional statistical methods use predefined rules that require prior knowledge, whereas ML aims to infer potentially unknown relationships between features from known examples, allowing the analysis of diverse data types frequently seen within healthcare [99].

Spectroscopic liquid biopsy data in particular is highly multi-modal, in that it analyses multiple biomarkers simultaneously, which is one of its benefits as it can identify interactions between biological ecosystems. ML is ultimately required to reveal these hidden interactions between the tumour and the body and relate them to cancer diagnosis [103]. Unlike genomics data, spectroscopic data has been shown to illustrate both tumour and non-tumour derived information. The performance of ML models heavily matters on the features used to train the model. Genomics data, such as the analysis of ctDNA, only looks at information directly related to the tumour, of which there is little present during the early stages. However, the body’s immune response is altered even at stage I/II, and so the analysis of non-tumour derived information is key to ML models being able to perform early detection.

Previously, the benefits of ML were reserved for industries that could afford the computational systems, such as space agencies and research organisations [104]. However, most ML methods are available as off-the-shelf software tools and can now be even used on personal computers, making it accessible to most. This has allowed the advancement in ML research as well as increasing applicability to the clinic, with most NHS hospitals using very primitive computer systems [4].

Although one of the requirements for complex ML models is large training datasets, there are methods available to overcome lack of available data, such as combining multiple models, which is common within the liquid biopsy field. Simple ML models can cope with small datasets, so it is possible to combine multiple simple models using model averaging. Creating this ensemble of models allows the information extracted from each model to be combined to create a more accurate prediction. Models can also be combined using Bayesian model averaging to give a weighted average prediction based on each of the individual model’s uncertainties, as well as stacking models which takes the output of a number of individual models and feeds it into a second-level model [45].

Another method for overcoming small dataset sizes and improving model performance is the use of generative models for data augmentation in which new synthetic data can be created to use for model training. These techniques have been successfully used within cancer diagnostics [105,106], and for the analysis of other spectroscopic data [107,108,109].

Overestimation has proven to be a problem among not only proof-of-concept studies, but also among inadequately designed validation studies, and should be overcome and avoided as much as possible. The first step should always be to utilise diverse training sets from multiple patient populations and clinical settings to ensure the model learns the features of these different patient demographics to improve the cancer diagnostic performance. However, where this is not possible during model development, methods such as transfer learning can also be used to improve the applicability of a model to different diagnostic scenarios [101].

## 4. From Lab to Clinic

To develop a ML spectroscopic liquid biopsy suitable for the clinic, numerous studies have to be carried out to determine if the test is going to be successful. These studies are broadly split into two categories: proof-of-concept and clinical validation studies.

### 4.1. Proof-of-Concept Studies

Proof-of-concept studies are the first stage in the development of any spectroscopic liquid biopsy. They are usually small-scale studies whose purpose are to demonstrate that the test has potential. In the case of spectroscopic liquid biopsies, the study may be used to determine that ML can distinguish between a small set of cancer and non-cancer patients. Overall, they aim to provide evidence that the new technology can answer a particular clinical question, as well as begin to illustrate how a test may benefit a clinical pathway [110].

It is common for proof-of-concept studies to utilise much smaller datasets than large clinical trials. This is often to reduce costs during the early discovery phase of development. However, when using spectral features as input to ML models, the number of features often match or exceed the number of training samples. This can often lead to overfitting and therefore extra steps need to be taken to mitigate these effects during early development, such as feature selection [45].

Small validation sets used during proof-of-concept studies are common but have often been criticised. Fadlelmoula et al. reviewed studies that used FTIR spectroscopy with ML for blood analysis. All the cons listed for each of the studies surround the idea that they require more data or a more diverse range of data [69]. Even if early-stage proof-of-concept studies wished to increase dataset sizes, large datasets from techniques that use human biospecimens, e.g., blood serum or plasma, are notoriously hard to come by compared to datasets acquired from electronic health records [70].

External test sets are vital to determine if the model can generalise to all aspects of a patient population and identify the limitations of the model. However, the cancer prevalence is often higher in the selected validation sets than it would be in the true clinical environment.

Collaboration and data sharing between researchers and companies could make the acquisition of validation datasets much more efficient. Having larger training sets measured on multiple systems in multiple locations would allow an ML model the opportunity to learn to ignore the noise associated with instrument variation to avoid overfitting [4].

Proof-of-concept studies, especially during the preliminary stages of test development, are predominantly retrospective, where the disease status of the patient is already known. However, prospective studies allow the analysis of cost and the effect on the patient that cannot be determined using retrospective tests alone [111]. Even though there are many liquid biopsy tests that show promise in retrospective cohorts, prospective testing is essential to assess clinical performance and utility [112].

One of the reasons for this lack of clinical utility is something known as spectrum bias, which is where a diagnostic test is studied in a different environment to the intended use population. An example of this is when retrospective studies collect cancer patients that already have a cancer diagnosis. This causes an overestimation in sensitivity as patients already with a diagnosis are most likely symptomatic and more likely to be at an advanced stage. By collecting prospectively, cancer patients can be discovered before symptoms and at an earlier stage, as well as being a more accurate representation of the target population the test would be used in [113].

Prospective studies are also useful as a comparison of current diagnostic methods. If a spectroscopic liquid biopsy is to be used to aid a clinician in diagnosis and treatment pathways, then the prospective study should include examples of decisions made with and without the liquid biopsy to represent the advantage of implementation [12,114]. However, the price per patient is substantially more during a prospective study compared with a retrospective study, often making the trials unattainable to academic research groups without the financial capabilities [115].

### 4.2. Clinical Validation

Once a liquid biopsy test had undergone multiple proof-of-concept tests, ideally both retrospective and prospective, the test should be clinically validated to fully determine its real-world use. Although not required currently for all regulatory pathways, clinical validation is vital to determine clinical utility, i.e., improved clinical outcomes [116].

There are three properties a liquid biopsy should hold in order to be a successful test. Both analytical validity, which is if the technique can accurately and reliably measure the analyte of interest, and clinical validity, which is if the test as a whole can accurately and reliably measure the clinical feature of interest, can be determined during proof-of-concept studies [116]. However, clinical utility, which determines the overall usefulness of a test, requires formal clinical validation.

Many current liquid biopsies can successfully demonstrate clinical validity, but few show clinical utility [117]. Large-scale clinical trials are required which involve the recruitment of patients from multiple populations across various clinical sites and comparing them with current diagnostic pathways [111]. However, the expense and time required to carry out these utility studies are out of the capabilities of most researchers and small companies, so are often not feasible [112].

## 5. Regulation

Once a liquid biopsy has sufficiently shown analytical validity and clinical validity and utility, the next stage is to achieve regulatory approval for clinical use and commercialisation. The regulatory requirements differ depending on the country of deployment, as well as how the test is used in practice.

### 5.1. Current Regulations for Liquid Biopsies

Current regulations for medical devices, In Vitro Diagnostic (IVD), and medical devices that utilise AI share similarities and differences between the US, UK, and EU. Although all regulators aim to ensure analytical and clinical validity as well as clinical utility, the lack of concrete regulation surrounding AI for IVDs is universal.

As of the passing of the Medical Device Amendments (MDA) in 1976, all medical devices have fallen under the regulation of the Food and Drug Administration (FDA) in the US. The overarching umbrella of medical devices includes IVDs when used for the diagnosis of disease [118]. If an IVD is manufactured and used by multiple laboratories, it must undergo regulatory approval processes, both pre- and post-market, to demonstrate patient safety and clinical effectiveness [12]. The level of regulatory processes required depends on the risk class assigned to the IVD, with low-, moderate-, and high-risk devices corresponding to class I, II, and III, respectively. Most class I devices are exempt from pre-market review, but the majority of IVDs require FDA authorisation before becoming commercially available [119].

There are various types of FDA approval processes. The least rigorous of the approval processes is a 510(k) premarket notification, which is used for medium-risk devices that are equivalent to devices already on the market. The equivalent to this, but requiring more evidential data than a 510(k) submission is a De Novo submission, which is also used for moderate risk devices but is instead used for novel devices. The most rigorous of FDA approval pathways is Premarket Approval (PMA) which is required for high-risk, novel device and requires extensive clinical trial data to demonstrate efficacy.

An exception to this are Laboratory Developed Test (LDT)s, which involve the manufacture and use of an IVD within a single laboratory. The FDA generally exercise enforcement discretion on LDTs and are instead certified under the Clinical Laboratory Improvement Amendments (CLIA) of 1988 to meet regulatory standards for clinical laboratories that utilise human samples for the purpose of prevention, diagnosis, or treatment of disease [119]. CLIA certification is presented only to the laboratory that was assessed and certified, whereas FDA approval allows an IVD to be used more broadly in different hospitals and clinics [12].

CLIA certification is less stringent that FDA regulation. CLIA certification focuses on the analytical validity of the use of the test in the chosen environment. The analytical validation of LDTs is also determined after the laboratory has already started testing, but does not allow the release of any test results until CLIA certification is achieved. On the other hand, FDA regulation requires analytical validation prior to marketing, as well as also addressing clinical validity and clinical utility [11].

The FDA at the time of the MDA release chose to implement enforcement discretion on most LDTs as they were generally low risk and primarily used for rare diseases. However, as LDT prevalence and complexity increased, the FDA were concerned for the lack of clinical utility demonstrated by these widely used test more frequently used for critical healthcare decisions. Therefore, in 2014, the FDA provided draft guidance documents to gain more oversight over the regulation of LDTs and assign similar risk categories to LDTs as with other medical devices, most likely placing IVDs into a high risk category [120]. Although this new regulation would ensure patient safety, critics stated the regulatory burden would stifle innovation due to the financial requirements that would be placed on laboratories to provide clinical utility evidence. The FDA countered this criticism by releasing examples of 20 LDTs that may have caused actual harm to patients due to the lack of regulation in place [121]. The FDA finalised this rulemaking in April 2024, meaning LDTs will now fall under FDA regulation. The phaseout of the enforcement discretion will be carried out over four years.

Another FDA regulatory pathway is its Breakthrough Device Designation Programme [122]. This allows devices that provide more effective treatment or diagnosis of life-threatening or irreversibly debilitating human disease or conditions to apply for breakthrough designation so that the FDA prioritises their regulatory applications, as well as having direct communication with the FDA to ensure clinical studies are designed optimally [123]. This programme aids current LDT tests to transfer from CLIA certification to full FDA approval quicker.

The FDA-equivalent in the EU, the European Medicines Agency (EMA), requires very similar regulations, excluding the differentiation of LDTs. Pre-2017, most IVDs fell under the In Vitro Diagnostic Medical Devices Directive (IVDD) in which IVDs were considered low risk and manufacturers could self-certify their test to claim it met the essential requirements to ensure patient safety [124]. In 2017, the In Vitro Diagnostics Regulation (IVDR) was introduced to require more stringent regulations and the requirement of accredited notified bodies to assess conformity. This resulted in almost 80% of the IVDs already on the market under the IVDD regulation having to undergo conformity testing from notified bodies due to the new risk class system in place, putting IVDs for cancer diagnosis into class C, one of the higher risk categories [125,126]. This ensures that IVDs are carrying out sufficient pre-market assessments and continuing post-market surveillance to retain the CE mark and remain on the European market [125].

Pre-Brexit, the UK also complied with the EU IVDR for the regulation of IVDs. However, as of 2021, the Medicines and Healthcare products Regulatory Agency (MHRA) took over responsibility for the oversight of all medical devices, including IVDs, and offers a UK Conformity Assessed (UKCA) mark as opposed to the CE mark offered by the EMA. The UKCA mark, however, remains based on the regulations from the EU IVDD, and therefore IVD manufacturers can self-certify their product by submitting a declaration of their conformity [127]. IVDs with a European CE mark were valid for market until 2023, but now all devices must register with the MHRA to become UKCA marked as well [128].

### 5.2. Current Regulations for AI/ML Medical Devices

As more IVD devices, particularly liquid biopsies, begin to utilise AI/ML to make diagnostic decisions, regulatory agencies have begun to put more stringent guidelines in place to monitor their usage and their effect on patients.

In the US, the FDA classifies IVDs that utilise ML as Software as a Medical Device (SaMD). These are classified similarly to a standard medical device in that it would fall into one of four classes dependent on level of risk, with a higher class corresponding to a greater level of regulatory requirements prior to marketing [129]. The majority of FDA approved ML-based SaMD consist of locked algorithms, in which new data is never added to the model to improve the diagnostic performance. If algorithms were to evolve as new patients were analysed, they may require follow-up FDA authorisation. This means that software engineers may be more inclined to use locked algorithms in order to reduce further costs and time associated with further regulatory approval [130].

However, despite the release of this guidance, there is still progress to be made in guidance for designing studies for AI medical device regulatory approval. A study conducted by Wu et al. looked into all AI medical devices approved by the FDA between 2015 and 2020. Of 130 devices investigated, 93 of them did not report multi-site sample collection and 126 of them only carried out retrospective studies for FDA evaluation [131]. Single-site, retrospective studies do not always demonstrate the real-life clinical use of an IVD. With 50% of all approved AI medical devices appearing in 2020 alone, the FDA understood the need for an improvement in how evaluation studies are carried out. Therefore, in 2021, the FDA aimed to increase the regulatory standards required for SaMD by publishing a document called Good Machine Learning Practice for Medical Device Development: Guiding Principles, which outlined 10 principles, such as the implementation of good software engineering and security, and ensuring that patients selected for studies are representative of the intended patient population. This was written in collaboration with Health Canada and the UK’s MHRA [132].

Previously, there was no specific regulatory pathway for AI medical devices in the EU similar to that provided by the FDA in the US. A study carried out by Muehlematter et al. showed that the majority of AI/ML-based medical devices that were approved in both the US and the EU achieved EU approval first, suggesting a less rigorous regulatory process [133]. This has led to safety concerns, with AI-based medical devices that were first approved in the EU reportedly being three times more likely to be recalled due to safety compared with those approved first by the FDA [134].

Therefore, in December 2023, the European Parliament reached an agreement on the AI Act, the first legal, comprehensive framework for the regulation of all AI. The new regulations will place AI devices into risk categories, from minimal risk to unacceptable risk. All products that would fall under the EU’s product safety legislation would be classified as high risk; this included medical devices such as liquid biopsies. High risk AI devices require adequate risk assessment and mitigation systems, and detailed documentation to describe the system to assess compliance. This would be in addition to the certification requirements described previously for the CE mark. This was formally published on 12 July 2024 and came into force on 1st August 2024. This regulation will be fully applicable by August 2026 [135,136].

The UK follows similar regulation to the US in that it classifies IVDs that use AI or ML as SaMD. They are also split into four classes dependent on risk and require a UKCA mark prior to market, regulated by the MHRA. In 2021, the MHRA published the Software and AI as a Medical Device Change Programme, which proposed an increase in the regulations surrounding the use of ML for IVDs [137]. It included plans for pre-market approval to ensure product safety and ongoing performance evaluation post-market based on the guidelines provided by the International Medical Device Regulators Forum (IMDRF) [129].

### 5.3. Currently Approved Liquid Biopsies

Despite the regulatory burden, there are a number of liquid biopsies that have been approved by agencies such as the FDA. The first liquid biopsy to be FDA approved was CellSearch^®^ by the Menarini Group in 2004 for metastatic breast cancer [138] and in 2007 and 2008 for colorectal [139] and prostate cancer [140,141]. It measures circulating tumour cells in plasma samples and the CellSearch System is 510(k) FDA-approved to use in clinics.

In 2016 two further liquid biopsy tests were approved by the FDA for clinical use. In June 2016, the Epi proColon^®^ test by Epigenomics was PMA approved for screening of colorectal cancer. It measures the level of methylated *SEPTIN9* ctDNA in patient plasma samples, which are found at low levels in healthy patients. It is routinely used for colorectal cancer screening for patients who cannot or choose not to partake in other screening tests, such as colonoscopies or stool-based tests [142]. Although it achieves a lower specificity when compared with stool-based tests, it has been shown to enhance colorectal screening participation due to the simplicity of a blood-draw compared to stool collection [143]. There is still a gap, however, for other blood-based biopsy tests to improve both adherence and sensitivity.

The second of these two tests to be PMA approved by the FDA in 2016 was the cobas^®^ EGFR Mutation Test by Roche Diagnostics, which analyses the mutations in the EGFR gene in patient plasma samples, and is a companion diagnostic for non-small-cell lung cancer [144]. A companion diagnostic is a test used to match a patient to a specific drug or therapy, or to monitor that drugs efficacy. The test takes only 4 h to return results and patients are only referred for a tissue biopsy if they return a negative result, which in turn reduces the number of invasive tissue biopsies [145].

These tests demonstrate the use of liquid biopsies for measuring a single biomarker or genetic mutation. However, in 2021, the FDA PMA approved two liquid biopsy tests that measure multiple genetic changes in plasma samples: Guardant360^®^ CDx (Guardant Health) [146]. and FoundationOne^®^ Liquid CDx (Foundation Medicine) [147]. Both analyse multiple genes in patient plasma samples for treatment guidance of non-small-cell lung cancer, with the FoundationOne Liquid CDx also providing treatment guidance for colorectal, prostate, and breast cancer. They both differ from previous FDA-approved tests in that they are approved as single-site tests, so the test can only be carried out in an FDA-approved laboratory [148].

All FDA-approved liquid biopsies involve the identification of genetic markers, e.g., ctDNA and cfDNA. Despite the promising research, there are still no spectroscopic techniques or ML-based methods approved by the FDA for diagnostic tests. One of the tests currently at the pre-approval stage is an early colorectal cancer detection test from Freenome, which analyses multiple components within a patient blood sample, including cfDNA, proteins, and biomarkers. It takes these components and combines them with ML techniques for the early detection of colorectal cancer and advanced adenoma [68]. In the latest results released for their PREEMPT CRC^®^ study, in which 27,000 patients due to undergo screening colonoscopy were recruited prospectively, the test was able to achieve 79.2% sensitivity for colorectal cancer with 91.5% specificity for patients with no, or non-advanced, colorectal neoplasia. However, they were only able to achieve a 12.5% sensitivity for advanced adenomas, which are considered a precursor to colorectal cancer [149]. This test is currently undergoing clinical validation [150].

Another liquid biopsy test that utilises ML but is yet to undergo regulatory approval is a Multicancer Early Detection (MCED) test from Exact Sciences. This test was originally named CancerSEEK and was developed by Thrive, and now acquired by Exact Sciences under the new name, Cancerguard™ [151,152]. It is a MCED test that analyses methylation and protein biomarkers of blood samples and combines them with a ML classifier to diagnose 21 different cancers. The most recent results of their ASCEND 2 study, which was a prospective study consisting of just over 6000 patients, were presented showing a 50.9% sensitivity at 98.5% specificity for all cancer types. This increased to 54.8% sensitivity when the cancers with screening protocols already in place were removed (excluding breast, prostate, cervix, colon, and rectum), and further increased to 63.7% when considering the most aggressive cancer types (pancreas, esophagus, liver, lung and bronchus, stomach, and ovary) [153]. Although this shows potential for cancer types without current screening programmes, the stage I and stage II sensitivities were only 15.4% and 38.0%, respectively, across all cancer types. Although CancerSEEK received Breakthrough Device Designation by the FDA in 2019, Cancerguard is still at the clinical validation stage [150].

Another MCED test in development that utilises ML is the Galleri^®^ test by GRAIL. This technology analyses more than 100,000 methylation sites in cfDNA to screen for more than 50 cancer types [154]. In their clinical validation study, the test was able to achieve 51.5% sensitivity at 99.5% specificity. However, much like CancerSEEK, the test was only able to achieve sensitivities of 16.8% and 40.4% for stage I and stage II cancers, respectively [155]. This test has also received Breakthrough Device Designation by the FDA, but unlike CancerSEEK, it is commercially available as an LDT [150]. The progression of these ML-based liquid biopsies demonstrate the potential of this technology in clinical practice.

## 6. Future Requirements and Predictions

As a number of liquid biopsies that utilise ML begins to increase, the need for standard, consistent evaluation guidelines is imperative to determine whether incoming ML models are relevant and beneficial to patients [14]. Not only should standard performance metrics, such as AUC, sensitivity, specificity, etc., be reported, but the usability, robustness, explainability, and proof of clinical impact should also be noted [156]. Performance metrics alone cannot compare two diagnostic tests that have different end uses. For example, if one liquid biopsy was developed to be used for cancer screening in a predominantly healthy population, and another was developed to be used only for those at a greater risk of cancer, the different probability thresholds would be chosen for each of these models. The former will tune the probability threshold to optimise specificity and reduce the number of false positive results, whereas the latter will optimise sensitivity to ensure that the maximum number of patients with cancer are caught early. Comparing the sensitivity or specificity of these two tests would not truly represent the impact on patients.

This difference in liquid biopsy application demonstrates the need to identify where a test should be used in the clinical pathway. During the early stages of test development, it would be useful for proof-of-concept studies to report performance metrics at multiple probability thresholds to identify where the test would be best suited. This would allow cost-effectiveness analysis to be carried out for a range of probability thresholds to determine the effects on health outcomes and health service costs by introducing the test at a certain clinical stage [157].

Although reporting general performance metrics can give an idea of how the model is performing, taking into account disease prevalence is important to determine the clinical applicability of the liquid biopsy test. Combining the sensitivity, specificity, and cancer prevalence produces a post-test probability. For example, if we proposed that the prevalence of cancer was 1% (which is an over-estimation of a general, healthy population) and we had an MCED test that has a sensitivity and specificity of 99%, this test would only achieve a post-test probability of 50%, which means half of the samples with a positive result would be a false positive [11]. This demonstrates why it is important for studies to report post-test probabilities in relevant populations to determine clinical validity of the liquid biopsy test.

Regarding spectroscopic liquid biopsies, the availability of larger datasets will be key in the progression towards regulatory approval. Additionally, the latest deep learning methods will only reach their full potential when datasets are sufficiently large enough for them to perform better than ML and to avoid overfitting [112].

For the uptake of ML with liquid biopsies to increase in the near future, the interpretability of the models must improve. The black-box effect of most ML models causes a lack of trust from clinicians as there is no biological understanding of how the model is making its predictions [12]. Not only would this increase the trust in ML models, it also has the potential to advance the understanding of cancer biology [158]. Although complex models associated with deep learning can never be fully elucidated, the predictions can be explained by looking at the contribution features have towards predictions [159].

Although there are many examples of ML combined with liquid biopsies successfully predicting patient samples in the literature, the generalisability of these tests needs to be proven to progress from research laboratories to the clinic. Using single-hospital datasets to test ML models does not always represent how the model will perform when faced with real data collected from multiple sites; this is why multi-centre studies are required to establish robust analytical and clinical validity [11,160]. Although small, retrospective studies are useful during the development stage, larger, prospective studies are needed to ensure the medical utility and to give a clearer picture of how the ML model might perform when applied in clinical care [161].

In terms of AI/ML regulation, the EU updated the regulation for AI medical devices to match the US FDA regulations. However, the UK is still to align their strategy, with AI regulations still in the proposal stages. An alignment of regulations worldwide will aid IVDs that utilise ML to reach the clinic with document requirements only having to be developed once for multiple approval applications. It is important that ML-based liquid biopsies are held to high regulatory standards to demonstrate their reliability for patients and clinicians [12].

Although improved AI/ML regulations ensure patient safety and trustworthiness in the models, these regulatory processes do not favour evolving algorithms, rather locked algorithms. Adapting the current standard for both pre- and post-market reviews are imperative to encourage development of algorithms to enhance their performance by allowing them to continuously learn from new data [123,160].

## 7. Conclusions

To conclude, ML has been used extensively alongside liquid biopsies in the research space, particularly within the field of spectroscopic liquid biopsies. With expensive and invasive screening tests being the only option for most patients to receive a cancer diagnosis, there is an urgent need for a more convenient method for diagnosis, particularly early detection of many cancers, which spectroscopic liquid biopsies can provide.

Utilising AI techniques alongside spectroscopic liquid biopsies has presented many opportunities, including the ability to learn from highly complex spectroscopic data to uncover the hidden tumour and body interactions and relate them to cancer diagnosis. There is also the benefit of being able to combine models and apply data augmentation techniques to overcome small dataset sizes that are often inevitable in cancer diagnostic studies. However, there are inherent challenges that go along with these advantages. ML models, particularly DL models such as neural networks, lack interpretability due to their black-box effect. Even though models may be effective at cancer diagnosis, the difficulty in understanding the underlying relationships in the data and how predictions were made make the implementation of ML models in the clinic challenging.

Many proof-of-concept studies have been conducted; however, the small test sets used to validate the models, often collected from single sites, do not represent the real-world population the test would be used on, causing the test to not generalise well to unseen data during clinical validation. Therefore, many spectroscopic liquid biopsies have demonstrated analytical and clinical validity, but very few have demonstrated clinical utility with the lack of large-scale, prospective trials from multiple populations across multiple clinical sites.

The FDA, and more recently the EMA, have imposed stricter regulations on the use of AI for IVDs with the purpose of increasing patient safety. Although this is the case, it provides another consideration for ML liquid biopsies before marketing for clinical use, as well as preventing the adaptation and evolution of ML algorithms as more data is made available. All regulatory bodies will need to adapt to favour these evolving algorithms, to enhance their performance and ultimately improve cancer diagnostic capabilities.

Overall, to date, there are no spectroscopic liquid biopsies, with or without ML, currently approved for clinician and patient use. Much work still needs to be carried out surrounding increasing the explainability of ML models and the use of large, diverse validation sets to prove their clinical utility. ML spectroscopic liquid biopsies have demonstrated their ability to diagnose cancer, even at early, asymptomatic stages. However, expansive validation sets and detailed regulatory requirements are needed to ensure implementation is at the forefront to transfer these findings from an academic setting to the clinic so that they have an impact on patients to improve survival and quality of life [162].

## Figures and Tables

**Table 1:** Descriptions of various spectral preprocessing techniques.

Preprocessing	Brief Description	Benefit	Example Algorithms and References
Spectral truncation	Also known as spectral cutting, removes regions of the spectrum to leave only purpose-relevant bands.	Removes noise from the spectrum and reduces dimensionality.	[71,72].
Baseline correction	Baseline of spectrum is fitted with a function and the new fitted baseline subtracted from the other spectral baselines.	Remove slopes in the baseline caused by interference and background effects to effectively compare peak intensities.	First and second derivative, rubberband, and polynomial [73,74].
Smoothing	Reduces the resolution of spectra by fitting a function to a pre-defined window of spectral points.	Reduces/removes spectral noise to enhance the biologically-relevant peaks.	Wavelet denoising, Savitzky-Golay filtering, and local polynomial fitting with Gaussian weighting [53,73,75,76].
Normalisation	Normalise spectra to allow the spectral intensities to be on the same, or similar, scales.	Allows multiple spectra to be effectively compared to each other.	Scaling of each spectrum between 0 and 1, vector normalisation, normalisation to Amide I band [73].
EMSC	Uses a reference spectrum and polynomial functions to correct baseline slopes and spectral noise.	Corrects additive baseline effects, multiplicative scaling effects, and interference effects.	[77].

## Data Availability

Not applicable.

## Abbreviations

**AI**	Artificial Intelligence
**ATR**	Attenuated Total Reflectance
**AUC**	Area Under the Curve
**cfDNA**	Cell Free DNA
**CLIA**	Clinical Laboratory Improvement Amendments
**CT**	Computed Tomography
**ctDNA**	Circulating Tumour DNA
**CV**	Cross-Validation
**DL**	Deep Learning
**EMA**	European Medicines Agency
**EMSC**	Extended Multiplicative Signal Correction
**FDA**	Food and Drug Administration
**FN**	False Negative
**FP**	False Positive
**FTIR**	Fourier Transform Infrared
**GPU**	Graphical Processing Units
**IMDRF**	International Medical Device Regulators Forum
**IR**	Infrared
**IVD**	In Vitro Diagnostic
**IVDD**	In Vitro Diagnostic Medical Devices Directive
**IVDR**	In Vitro Diagnostics Regulation
**LDT**	Laboratory Developed Test
**MCED**	Multicancer Early Detection
**MDA**	Medical Device Amendments
**MHRA**	Medicines and Healthcare products Regulatory Agency
**ML**	Machine Learning
**MRI**	Magnetic Resonance Imaging
**NN**	Neural Network
**NPV**	Negative Predictive Value
**PLS**	Partial Least Squares
**PMA**	Premarket Approval
**PPV**	Positive Predictive Value
**RF**	Random Forest
**ROC**	Receiver Operating Characteristic
**SaMD**	Software as a Medical Device
**TN**	True Negative
**TP**	True Positive
**UKCA**	UK Conformity Assessed
